# Diffuse Axonal Injury: Clinical Prognostic Factors, Molecular Experimental Models and the Impact of the Trauma Related Oxidative Stress. An Extensive Review Concerning Milestones and Advances

**DOI:** 10.3390/ijms221910865

**Published:** 2021-10-08

**Authors:** Mauro Palmieri, Alessandro Frati, Antonio Santoro, Paola Frati, Vittorio Fineschi, Alessandro Pesce

**Affiliations:** 1 Neurosurgery Division, A.O.U. “Policlinico Umberto I”, Human Neuroscience Department, “Sapienza” University, Viale Del Policlinico 155, 00161 Rome, Italy; alessandro.frati@uniroma1.it (A.F.); antonio.santoro@uniroma1.it (A.S.); 2 IRCCS “Neuromed”, Via Atinense 18, 86077 Pozzilli, Italy; 3 Department of Anatomical, Histological, Forensic and Orthopaedic Sciences SAIMLAL, “Sapienza” University, Viale Regina Elena 336, 00185 Rome, Italy; paola.frati@uniroma1.it (P.F.); vittorio.fineschi@uniroma1.it (V.F.); 4 Neurosurgery Division, Santa Maria Goretti Hospital, Via Lucia Scaravelli, 04100 Latina, Italy; ale_pesce83@yahoo.it

**Keywords:** traumatic brain injury, diffuse axonal injury, oxidative stress, reactive oxygen species, biomarkers

## Abstract

Traumatic brain injury (TBI) is a condition burdened by an extremely high rate of morbidity and mortality and can result in an overall disability rate as high as 50% in affected individuals. Therefore, the importance of identifying clinical prognostic factors for diffuse axonal injury (DAI) in (TBI) is commonly recognized as critical. The aim of the present review paper is to evaluate the most recent contributions from the relevant literature in order to understand how each single prognostic factor determinates the severity of the clinical syndrome associated with DAI. The main clinical factors with an important impact on prognosis in case of DAI are glycemia, early GCS, the peripheral oxygen saturation, blood pressure, and time to recover consciousness. In addition, the severity of the lesion, classified on the ground of the cerebral anatomical structures involved after the trauma, has a strong correlation with survival after DAI. In conclusion, modern findings concerning the role of reactive oxygen species (ROS) and oxidative stress in DAI suggest that biomarkers such as GFAP, pNF-H, NF-L, microtubule associated protein tau, Aβ42, S-100β, NSE, AQP4, Drp-1, and NCX represent a possible critical target for future pharmaceutical treatments to prevent the damages caused by DAI.

## 1. Introduction

Traumatic brain injury (TBI) is a condition burdened by an extremely high rate of morbidity and mortality and can result in an overall disability rate as high as 50% in affected individuals [1]; in the near future, it will become the third leading cause of permanent disability and mortality worldwide [2]. TBI is a frequent cause of traumatic diffuse axonal injury, realizing a condition called diffuse axonal injury (DAI). Because of its incidence and its economic impact on national health systems [3,4], the importance of identifying clinical prognostic factors for diffuse axonal injury (DAI) in traumatic brain injury (TBI) is commonly recognized as critical [5]. More specifically, initial GCS, duration of the loss of consciousness, blood oxygenation, and blood pressure are some of the several prognostic factors that have been investigated in order to understand their impact on the clinical and neurological outcome of DAI in TBI [5,6,7,8,9]. Furthermore, new light has been recently shed over the possible influence of the mechanisms managing trauma-related oxidative stress in determining the severity of the clinical phenotype of the TBI: the imbalance between the production and removal of reactive oxygen species (ROS), the release and activation of pro-inflammatory cytokines, and modifications in calcium metabolism are just a few relevant examples.

The aim of the present review paper is to encompass and analyse the most recent contributions from the relevant literature in order to understand the weight of each single prognostic factor in determining the severity of the clinical syndrome associated with diffuse axonal injury.

## 2. Hypoxia and Blood Pressure

The presence of hypoxia (measured by SpO2: SpO2 < 90%; measured by PaO2: PaO2 < 60 mmHg) and hypotension (measured by blood systolic pressure < 90 mmHg) is a physiological change after TBI and is associated with a major fatality rate [10]. In fact, preserving airway patency after a traumatic injury is recognised as a positive prognostic factor [11]. The relative inability to increase cerebral blood flow in response to hypoxia and hypotension after a traumatic injury exposes the injured brain to a secondary ischemic insult [12]. In the study presented by Manley et al. [12], it is reported that in patients with hypoxia alone, the mortality was 28%, while the presence of both hypoxia and hypotension increased the mortality to 57%. These findings are confirmed in other studies [13,14,15], and the association between hypoxia and hypotension with increased mortality has also been demonstrated in children [16]. Moreover, repeated episodes of hypotension have a negative impact on survival [12]. Regarding hypertension (blood systolic pressure ≥ 160 mmHg), Viera et al. [10] highlight the impact of elevated levels of systolic blood pressure on intracranial pressure and cerebral edema even though hypertension may be a physiological response to reduced cerebral perfusion. Therefore, the decision to treat hypertension should be made carefully since lowering the blood pressure could exacerbate cerebral ischemia itself [17]. In conclusion, according to the latest guidelines concerning the management of severe traumatic brain injury [18], the value of 90 mmHg for systolic blood pressure and of 60 mmHg of peripheral blood oxygenation are considered to be the thresholds to define hypotension and hypoxia, respectively, representing a statistically significant factor influencing the outcome.

## 3. Glycemia

Changes in glycemia levels in severe TBI are related to the increased blood levels of catecholamines caused by the sympathetic hyperactivity after the traumatic injury [19,20]. Hyperglycemia induces lactic acidosis, which extends secondary neuronal damage [21]. On the other hand [21], it is commonly known that hypoglycaemia induces neuronal necrosis, especially in the caudate, putamen, and hippocampus [21]. Liu-De Ryke et al. [22] analysed the association between glycemia in the first 5 days and mortality after TBI in a cohort of 380 patients. The glucose cut-off values of 7.5 mmol/L for admission and 8.89 mmol/L for the day 1 peak were demonstrated to be effective predictors of mortality [22]. More specifically, a significant difference in terms of survival was detected for the threshold of the 8.89 mmol/L day 1 peak. In fact, on day 5, 96% of the patients with glucose blood levels <8.89 mmol/L survived versus the 75% of those with glucose blood levels >8.89 mmol/L. They also highlighted the correlation between high levels of blood glucose and trauma severity. Moreover, in the same study, patients with glycemia <3.33 mmol/L showed a higher mortality rate than those with a glucose blood level >3.33 mmol/L. Regarding hyperglycemia, other studies [10,23,24,25] have reported that increased levels of blood glucose correlate with poor outcome. Up until now, a common threshold for hyperglycemia has not been identified; thus, modern guidelines [18] do not include pharmacological interventions for changes in glucose blood levels. 

## 4. Pathological Anatomy and Morphologic Findings

The focal lesions caused by a severe form of DAI can be macroscopically identified postmortem [26]. In patients who do not survive, the lesions usually appear as hemorrhagic, while the identification of the lesion after weeks or months could be challenging due to the presence of gliosis [26]. In the series presented by Adams et al. [26,27] DAI has three distinctive structural features:

1. Diffused supratentorial damage to axons (grade I).
2. A focal lesion in the corpus callosum (grade II).
3. A focal lesion or multiple lesions in the rostral brain stem (grade III).

**Figure 1:**
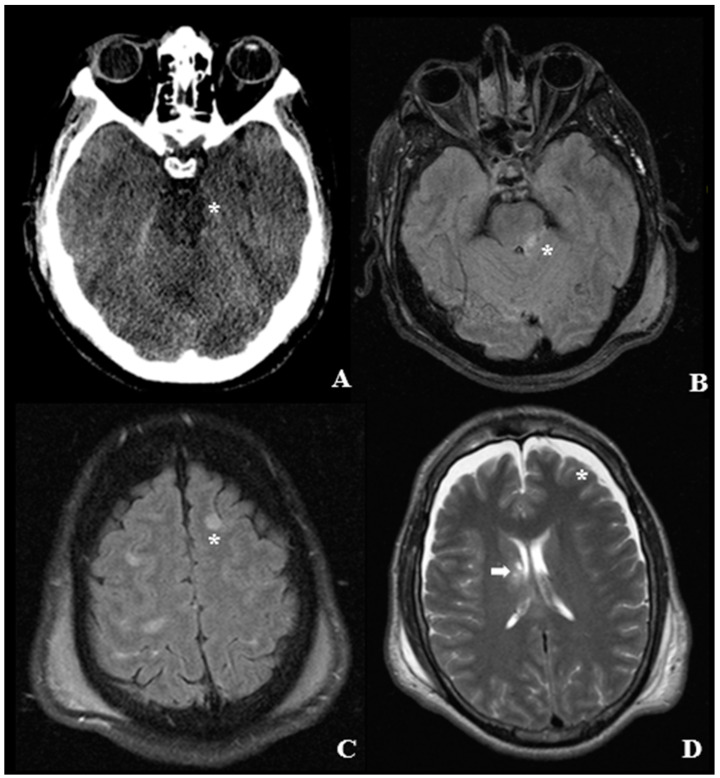
Radiological findings in a case of DAI—(**A**) First Computed tomography (CT) scan disclosing the absence of Ambiens Cistern and a slight hypodensity of the transition zone between Mesencephalon and Pons (asterisk). (**B**,**C**) Flair sequence late MRI disclosing the diffuse involvement of the subcortical with matter and of the Brachium Pontis (asterisks). (**D**) T2 weighted MRI scan disclosing the late atrophic changes of the Supratentorial cerebrum, with a bilateral Subdural Hygroma (asterisk). The arrow highlights a neural injury at the transition between the body of the Caudate Nucleus and the Internal Capsule.

Furthermore, grade II and III lesions have typical localization. In fact, lesions in the corpus callosum typically occur in its inferior part and to one side of the midline, while lesions on the rostral brainstem typically localizes themselves in the dorsolateral quadrant or in the quadrants adjacent to the superior cerebellar peduncles [27,28], Figure 1. However, the diagnosis of DAI can only be confirmed through histological studies since axonal damage cannot be identified during macroscopical examination [28].

In order to diagnose DAI, swollen axonal varicosities and axonal bulbs [29] must be detected during microscopic examination [30]. According to modern studies [31], the two main characteristics that must be identified are the presence of diffuse/multifocal axonal damage in the white matter and this axonal damage being widespread in many brain regions, at least one of which should be located above the tentorium and one should be located below the tentorium. The morphological aspects of DAI are also influenced by the time span of interlude between the beginning and resolution of coma; in fact, the evolution of microscopical damage has been described since the 1980s [26]. In order to classify the severity of axonal injury according to microscopical findings, Gennarelli et al. [32] have divided different types of lesions into three grades of severity:

Grade 1: There are scattered axonal retraction balls in the parasagittal white matter of the cerebral hemispheres, the corpus callosum, the brain stem, and, less commonly, the cerebellum.

Grade 2: In addiction to axonal damage in the white matter of the cerebral hemisphere, there is a focal lesion in the corpus callosum.

Grade 3: In addition to axonal damage in the white matter of the hemispheres, there are focal lesions present in the dorsolateral quadrant of the rostral brain stem and the corpus callosum.

## 5. Time to Recover Consciousness

The impact of the severity of DAI on the recovery of consciousness in patients with TBI is well recognized [8,9]. More specifically, a correlation has been observed between the mean time interval to recovery of consciousness in patients with DAI and the degrees of brain injuries according to the classification proposed by Gennarelli et al. [5,32]. Based on the findings of the studies [33,34] that showed the impact of magnetic resonance imaging (MRI) findings and DAI outcome, Park et al. analysed data concerning the severity of lesions identified with MRI with the time to recover consciousness. The results outlined a faster recovery time for patients with a grade I and II injury despite grade III lesions, describing a time span of weeks for lesions involving cerebral white matter and the corpus callosum and for months for injuries located in brain stem. However, a universally accepted classification for time to recover consciousness has not been identified yet regarding patients with DAI.

## 6. Severity of Trauma

The Injury severity score (ISS) and the New Injury Severity Score (NISS) represent two universally accepted scales in the evaluation of the severity of trauma [35,36] and, specifically for head trauma, the Maximum Abbreviated Injury Scale (MAIS) [37] represents a more accurate tool for the evaluation of TBI. The ISS and NISS scales divide the body into different regions, and for each of them, a score from 1 to 6 is given on the basis of the severity of the lesion in that particular anatomical area [35,36]. Then, the final score is useful in order to predict mortality, morbidity, and hospitalization length after trauma. The MAIS represents a subscale of the ISS, and it represents the score of a single body region. A MAIS of 5 is related to a critical damage of the area of interest, and it relates to a poor outcome [10,37]. High scores in the ISS and NISS are related to a higher risk of poor outcome [10], and it has also been demonstrated that NISS can predict mortality better than ISS [38], even if there is scant literature on the application of NISS in DAI [10]. Furthermore, high scores in the three scales relate to dependence, which can be measured with the Glasgow Outcome Scale-Extended (GOSE) [39] 6 months after DAI [10]. The aforementioned scales take in account the clinical impact of possible concurrent lesions involving other organs or other collateral conditions, including intracranial bleeding, cranial base and vault lesions [40], and spinal cord or vertebral lesions. Moreover, it has been described that pre-existing conditions, namely other major comorbidities and aging-related changes to tissues and metabolism could worsen the injury caused by the trauma [30,40,41].

## 7. GCS

The Glasgow Coma Scale (GCS) [42] undoubtedly represents one of the most important neurological tools to assess the neurological performance of a patient affected by TBI. The final score of the scale ranges from 3 to 15, and it is obtained by the summation of the scores of the three subscales regarding eye response, verbal response, and motor response. A GCS score <8 represents a severe TBI, and in patient with DAI, lower GCS score levels at admission are related to higher mortality and dependence after trauma [10,25,43]. Moreover, higher levels of dependence and mortality are related also to lower GCS score levels after the withdrawal of sedation [10,44]. According to Skandsen et al. [45], GCS is correlated to outcome only if DAI happens in TBI, outlining the importance of diagnosing this kind of injury. Whereas the GCS score at admission is, according to a vast number of clinical trials, strongly associated with the prognosis of patients, it should be kept in mind that a great number of patients suffering from a TBI-related DAI (including DAI I and II) may acutely present with lower GCS scores (3–6), possibly not accurately discriminating the long-term prognostic classes of patients. The GCS score should therefore be interpreted in the context of the specific trauma of each single patient while also relating to the aforementioned biological parameters. 

## 8. Biomarkers and the Role of Oxidative Stress

Besides clinical prognostic factors, biomarkers related to the physiopathology of DAI represent a promising solution for defining novel strategies for the assessment of the severity of the injury and for pharmacological treatment [46]. In the process of axonal swelling and neural death, the mitochondrial permeability transition pore (mPTP) is a protein that is produced after a major accumulation of calcium in the mitochondria in response to the cellular exposition to reactive oxygen species (ROS) [47,48,49,50]. Therefore, the possible use of cyclosporin A, an inhibitor of mPTP, as a pharmacological option in the treatment of DAI is under investigation [51,52,53,54,55,56]. Buki et al. [51] conducted an experimental study on Sprague Dawley rats regarding the intrathecally administration of cyclosporin A 30 min before causing brain injury. The first result retrieved from this study shows that cyclosporin A preserves mitochondrial integrity in axons destined to be disconnected after the brain injury. According to the authors, the neuroprotection effect caused by cyclosporin A is due not only to the inhibition of mPTP but also to the reduction of calcium-induced, calpain-mediated spectrin proteolysis (CMSP) and neurofilament compaction (NFC), which are directly involved in the pathogenesis of axonal disconnection. In fact, in this experimental study [51], it was shown that the administration of cyclosporin A 30 min after brain injury resulted in a significant decrease in CMSP and NFC immunoreactivity in the corticospinal tracts and medial longitudinal fasciculi 24 h after injury. Similar findings have been described by Okonwko [52,53], who highlighted how mitochondrial damage leads to calcium-induced cytoskeletal modifications after traumatic axonal injury by specific antibodies that target spectrin proteolysis and neurofilament compaction. Immunoreactivity studies conducted in rats treated with cyclosporin A before axonal injury and in those that did not receive any premedication showed that the administration of cyclosporin A prior brain injury causes significative decreases in cytoskeletal injury (approximately 70%). Even though these promising results have been obtained in animal studies, there is still a lack of consistent evidence proving the effectiveness of cyclosporin A in humans. Aminmansour et al. [57] designed a randomized double-blind placebo-controlled study with 100 patients suffering from DAI. A total of 50 patients were treated with 5 mg/kg/24 h of cyclosporin A via a 250 mL dextrose water (DW) 5% solution (DW 5%) in the first 8 h after trauma, while the control group (n = 50) only received DW 5% in the same course. Serum values of complete blood count, blood urea nitrogen, creatinine, aspartate aminotransferase, alanine aminotransferase, and alkaline phosphatase were registered as well to assess the incidence of possible complications due to pharmacological treatment with cyclosporin A. The analysis of the GOSE score comparison between the two groups at 3 and 6 months after the trauma did not show significant differences. With the exception of a transitional elevation of blood urea nitrogen in the first 48 h and of white blood cell count at 12 h in the intervention group, no long-lasting significant differences were outlined regarding adverse effects. Similar results were obtained by other authors [58,59,60], suggesting that the administration of cyclosporine A still does not lead to any clinically relevant effects. 

Regarding cytoskeletal injury, the role of caspase and calpain in the cytoskeletal decomposition process has been investigated extensively [61,62]. In the proteolysis process induced by these enzymes, the neurofilament protein (NF) is interesting [63], and the accumulation of its subunits after TBI is currently being studied in order to identify predictive markers for outcome. Serum levels of the phosphorylated neurofilament heavy subunit (pNF-H) at 24 and 72 h after TBI [64] and the neurofilament light chain (NF-L) [65] protein serum level has recently been advocated as a potentially promising predictor of 12-month neurological outcome. More specifically, Shibahashi et al. [65] collected blood samples from 32 human subjects after brain injury, and they included patients with a GCS score of 13 or less upon admission and assessed outcome at 6 months using the same scale. The authors showed that the pNF-H serum levels that exceeded 240 pg/mL 24 h after trauma represented a significant predictive outcome at 6 months (*p* < 0.001). The PNF-H serum levels > 80 pg/mL 72 h after TBI correlated significantly with unfavorable outcome as well at 6 months (*p* < 0.01). Regarding NF-L, Ljungqvist et al. [66] measured acute serum concentrations of this polypeptide in nine patients affected by severe DAI by means of an ultrasensitive single molecule array (Simoa) assay and analyzed their correlation with clinical outcome and MRI diffusor tensor imaging (DTI) parameters at 12 months after TBI. The blood samples were collected 6 days after TBI, and the NF-L serum levels were measured in 22 age-matched healthy controls. The statistical analysis highlighted that the patients affected by DAI had a 30-fold increase of NF-L serum concentrations than healthy controls and that the level of NF-L correlated with the severity of DAI. More specifically, higher serum levels of NF-L were significantly associated with higher trace (R^2^  =  0.79) and lower fractional anisotropy (FA) (R^2^ =  0.83) DTI parameters and consequently worse clinical outcome. Even though these two biomarkers represent a promising solution for DAI assessment, they have been tested in small cohorts; therefore, further investigation must be conducted through large prospective trials to confirm their reliability. 

Other biomarkers that are under investigation as potential predictors of outcome in DAI and that are related to cytoskeletal injury are the glial fibrillary acid protein (GFAP) [66,67], microtubule-associated protein tau [68], and the amyloid β peptide (Aβ42) [69]. According to Bogoslovsky et al. [70], the combined measurement of the plasma levels of all three biomarkers could be useful in both acute and subacute phases of TBI. The study proposed by these authors was conducted on 34 patients affected by TBI with consequent DAI and a control group of 69 healthy volunteers. Plasma samples were collected 24 h and 30 and 90 days after the trauma. More specifically, the GFAP and microtubule-associated protein tau peaks were registered 24 h after injury, while the Aβ42 levels in the plasma reached their maximal concentrations at day 30. All three biomarkers maintained high blood values up to 90 days after the trauma. Moreover, Aβ42 levels after 30 days and GOSE measured at 180 after TBI showed a relationship after adjustment for age, and a weak correlation was outlined between Tau concentrations at day 30 and radiological injury severity. 

The calcium binding protein S-100β increases in the early phases of injury [71,72] although there are some limitations related to its usage. First of all, opposing evidence on the dependence of S-100β serum concentrations from the integrity and permeability of the blood–brain barrier (BBB) have been described [73,74]. In addition, the elevation of S-100β seems to be related to musculoskeletal injury in polytrauma patients [75], so the interpretation of high levels of S-100β in this kind of patient should be evaluated with caution even though the consequences of polytrauma damages on the serum dosage of this protein appear to be limited to the first 12 h due to its short half-life [76]. In conclusion, in the series presented by Pelinka et al. [77], the GFAP was more reliable than S-100β in discriminating between severe disability and a persistent vegetative state, while the data concerning mortality was demonstrated to be superimposable. 

High levels of neuron-specific enolase (NSE) are measurable in cerebrospinal fluid (CSF), but despite its ability to be measured from S-100β serum levels depends on the integrity of the BBB [78] even though conditions such as polytrauma should not influence the blood levels of NSE. Furthermore, the NSE half-life is significantly longer than S-100β, making its sampling more manageable. Thelin et al. [76] measured NSE and S-100β in 417 patients twice daily, and both biomarkers resulted in being independently correlated to long-term functional outcome. Moreover, S-100β appears to have higher precision as an outcome predictor in TBI and, consequently, a more reliable biomarker than NSE [76]. Evidence regarding pediatric TBI suggests that NSE could be a good predictor for Glasgow Outcome Score (GOSE) results [79].

The expression of the nuclear factor erythroid-related factor 2 (Nrf2) has recently been identified as a protective factor towards ROS induced by DAI [80]. Nrf2 is sensitive to redox reactions [72,81], and it regulates the expression of hemeoxygenase-1 (HO-1) by binding to antioxidant the response element (ARE) [82,83]. Moreover, Nrf2 can also neutralize the effects of carcinogens or ROS directly [84]. Ishii et al. [81] first reported that oxidized low-density lipoproteins (oxLDLs) and 4-Hydroxy-2-nonenal (HNE) derived from lipid oxidation induced the nuclear translocation of Nrf2 in murine peritoneal macrophages. The expression of Nrf2 causes the consequent upregulation of CD36, which represents a scavenger receptor in the macrophages, and its role in homeostasis and anti-inflammatory responses is also regulated by PPAR-γ. The evidence retrieved from this study focused its attention on the possible role of Nrf2 in oxidative stress mediated injuries, and Gao et al. [83] demonstrated its role in DAI. In fact, after DAI-related oxidative stress, Nrf2 is released from its inhibitor Kelch sample-related protein (Keap1) and binds to ARE inside the nucleus, causing the consequent expression of NAD(P)H:quinone oxidoreductase 1(NQO1) and HO-1 [83]. Gao et al.’s experiments confirmed that Nrf2 is increased in rats affected by DAI while it remains normally expressed in controls through Western Blot analysis (*p* < 0.01) at 6, 24, 28, and 72 h. Moreover, regarding the expression of both HO-1 and NQO1, analogue results were retrieved after RT-PCR analyses on rat brain stems after TBI, highlighting a time-dependent increase with a peak after 24 h, high expression until 72 h after injury, and a consequent gradual decrease. The comparison with the control group showed that these changes were constantly significant (*p* < 0.05). 

On the basis of these findings, Wu et al. [80] described that the expression of Nrf2 and, consequently, the HO-1 in cerebral microvessels is correlated to the progression of DAI, the severity of blood–brain barrier damage, and cell injury caused by ROS. Therefore, given its important role as an antioxidant agent, the authors highlighted how Nrf2 may represent a crucial molecular target in order to protect brain cells from ROS. In particular, they induced the expression of Nrf2 by means of an exogenous molecule administration. The role of sulphorafane (SFN) in inducing the expression of different antioxidant agents in human studies has been extensively described by Dinkova-Kostova et al. [85]. In this review, it was reported how after 2 weeks of daily consumption of 100µmol SFN, the mRNA levels of Nrf2 and HO-1 were upregulated in a dose-dependent manner. Therefore, Wu et al. [80] proposed the use of SFN in an animal model of DAI to induce Nrf2 expression and to consequently reduce DAI-related ROS and neuronal apoptosis. The authors divided 48 Sprague Dawley rats into four equally formed groups: the control group, the DAI group, the DAI group treated with phosphate-buffered saline (PBS), and the DAI group treated with SFN. Cell samples retrieved from all of the groups were then treated for 24 h with H202 and/or SFN after incubation. In vivo and in vitro analysis were conducted. The results showed that the DAI-related oxidative stress damages that were significantly more represented were those where the treatment with SFN was not performed, outlining the protective effects of SNF on programmed cells death due to axonal injury. Moreover, further analysis conducted on samples with and without Nrf2 defection showed that when Nrf2 is normally expressed, treatment with SFN is more effective in suppressing neuronal apoptosis and that Nrf2/HO-1 activation due to SNF is confirmed both in vitro and in vivo.

Several authors have advocated for a potentially critical role as a biomarker and a therapeutic target of the dynamin related protein-1 (Drp-1) and the reverse-mode sodium calcium exchanger (NCX) [86]. NCX modulates the Ca^2+^ intracellular influx on the ground of an exchange mechanism based on the intracellular Na^+^ concentration: under TBI-induced stress conditions, the abnormal glutamate release leads to an intracellular Na^+^ increase, thus reversing the activity of this exchange protein, leading to an accumulation of Ca^2+^ in the intracellular compartment [87,88,89], eventually determining a diffuse mitochondrial dysfunction resulting in the creation of ROS and the activation of endoproteases, phospholipases, and endonucleases [90], resulting in axonal degeneration and neuronal death. Drp-1 is a GTP-ase involved in mitochondrial fission [86]. These potential target proteins have been found to be expressed in the homo- and contralateral cortex and the hippocampus after experimental TBI at 1 h and 24 h after the TBI, with a progressive reduction and a return to the baseline levels within 5 days [86]. In order to test Drp-1 and NCX as possible therapeutic targets, Omelchenko et al. [86] used a microfabricated culture platform to create a model of in vitro DAI samples. The authors observed that Drp-1 upregulation was connected with TOMM20, a mitochondrial marker, in the proximity of injured axons. Similarly, NCX was clustered along the axonal processes in the control group, while in the post-stretch injury group, NCX localized to the axonal beads. Therefore, selective inhibitors of Drp-1, dynasore, and of NCH, SN-6, were tested immediately post-injury and every hour for 6 h. After examination, both dynasore and SN-6 showed protective effects in preventing increases of axons bead side damage after 6 h. However, a minor increase of post-traumatic axonal swelling was observed but only in the Dynasore treatment group. Regarding mitochondrial fragmentation induced by axonal strain, the authors compared the morphology of the axonal mitochondria before and 1 min after injury treatment with Dynasore and SN-6. The comparison with the control group highlighted that both drugs are effective in avoiding morphological and dimensional modifications, suggesting that NCX and Drp-1 are directly involved in the molecular cascade responsible for mitochondrial fragmentation [86]. 

Further analyses were conducted to assess the role of dynasore and SN-6 in preventing oxidative stress damage related to DAI, but only dynasore was able to attenuate H202-induced apoptosis and increase of ROS in all hippocampal regions. In conclusion, potential therapeutic agents such as dynasore and SN-6 have shown promising results in attenuating the stretch injury-induced swelling of axonal varicosities and mitochondrial fragmentation, thus expressing potential clinical significance as therapeutic agents in the clinical management of DAI. Moreover, Drp-1 could represent a specific molecular target for preventing oxidative stress injury related to DAI. 

Recent studies regarding aquaporin-4 (AQP4) have focused on the increase of its expression and subcellular relocation of this protein after hypoxia-induced cell swelling through a calmodulin-mediated mechanism [91,92]. AQP4 is the most common isoform of acquaporin proteins in the central nervous system (CNS), and it has been identified as the major regulator of water homeostasis through the control of the exchange of CSF with interstitial fluid [92]. Therefore, modifications in AQP4 expression are consequently related to the development of brain edema. More specifically, an increase of AQP4 activity in brain edema is caused by the upregulation of calmodulin when hypoxia-mediated injury occurs. Calmodulin directly binds the AQP4 carboxyl terminus, causing a conformational change and the consequent cell-surface location of AQP4, which leads to cell swelling due to a massive increase of intracellular water [91]. It has been described by Kitchen et al. [91] that trifluoperazine (TFP) inhibits the calmodulin-mediated cell location of AQP4 by directly binding to calmodulin in vivo by creating a crush model injury at T8 and a cortical stab injury in rodents. The consequent development of CNS edema can be prevented by the administration of TFP. In this study [91], the authors proved that in both the crush injury and in the cortical stab injury models treated with TFP, there is a significant difference in terms of the attenuation of CNS edema and improvement in electrophysiological, sensory, and locomotor function. Sylvian et al. [93] confirmed these findings by using a photothrombotic stroke model in rats. TFP (10 mg/kg) was administered subcutaneously at either 30 min before stroke or 1 h after stroke. A significant brain edema reduction was only observed in the group treated 1 h after the photothrombotic stroke, and TFP had not shown any effects on the investigated brain neurochemical biomarkers, electrolytes, or elemental homeostasis. This promising evidence suggests a possible future perspective for AQP4 inhibitors to be tested to treat CNS edema after TBI even though there is still a lack of studies in humans.

## 9. Conclusions

DAI is a leading cause of mortality and permanent morbidity worldwide. Despite numerous efforts, the clinical prognosis of the high-grade DAI remains, to date, in many cases, dismal. Multiple prognostic factors have been assessed in the last decade. Among those factors, the importance of early GCS, peripheral oxygen saturation, glucose serum levels, and blood pressure have confirmed their clinical significance in high-impact trials. The role of the oxidative stress in regard to the intracellular ROS balance and Ca^2+^ cellular metabolism is increasingly being investigated, with noteworthy preliminary results. Markers such as GFAP, pNF-H, NF-L, microtubule-associated protein tau, Aβ42, S-100β, NSE, AQP4, Drp-1, and NCX have demonstrated significant effectiveness in predicting clinical outcomes in animal studies, but, at present, their assessment in human studies is still confined to small case series groups and is not included in the guidelines for the management of TBI-related DAI. Regarding possible pharmacological agents that could control and reduce morphological and ROS damages related to DAI, SFN, and TFP, Dynasore and SN-6 have shown promising results in vitro and in animal studies; however, further investigations are needed to verify their safety and effectiveness though it has been demonstrated that the administration of cyclosporin A does not lead to any clinically relevant effects in humans.

## Data Availability

Not applicable.

## Abbreviations

TBI	Traumatic Brain Injury
DAI	Diffuse Axonal Injury
ROS	Reactive Oxygen Species
ISS	Injury Severity Score
NISS	New Injury Severity Score
MAIS	Maximum Abbreviated Injury Scale
GOSE	Glasgow Outcome Scale-Extended
GCS	Glasgow Coma Scale
mPTP	Mitochondrial Permeability Transition Pore
NF	Neurofilament Protein
pNF-H	Neurofilament Heavy Subunit
NF-L	Neurofilament Light Chain
GFAP	Glial Fibrillary Acid Protein
BBB	Blood–Brain Barrier
NSE	Neuron Specific Enolase
CSF	Cerebrospinal Fluid
Aβ42	Amyloid β Peptide
MRI	Magnetic Resonance Imaging
CT	Computed Tomography
NCX	Reverse-mode Sodium Calcium Exchanger
Drp-1	Dynamin Related Protein-1
Nrf 2	Nuclear factor erythroid 2-related factor 2
HO-1	Hemeoxygenase-1
ARE	Antioxidant Response Element
SNF	Sulphoraphane
Drp-1	Dynamin related protein-1
DTI	Diffuse Tensor Imaging
oxLDLs	oxidized low-density lipoproteins
HNE	4-Hydroxy-2-nonenal
NQO1	NAD(P)H:quinone oxidoreductase 1
Keap1	Kelch sample related protein
PBS	Phosphate-Buffered Saline
AQP4	Aquaporin-4
TFP	Trifluoperazine
CNS	Central Nervous System
